# Use of a Large Language Model to Identify and Classify Injuries With Free-Text Emergency Department Data

**DOI:** 10.1001/jamanetworkopen.2024.13208

**Published:** 2024-05-28

**Authors:** Giulia Lorenzoni, Dario Gregori, Silvia Bressan, Honoria Ocagli, Danila Azzolina, Liviana Da Dalt, Paola Berchialla

**Affiliations:** 1 Unit of Biostatistics, Epidemiology and Public Health, Department of Cardiac, Thoracic, Vascular Sciences and Public Health, University of Padova, Padova, Italy; 2 Division of Pediatric Emergency Medicine, Department of Women’s and Children’s Health, University of Padova, Padova, Italy; 3 Department of Environmental and Preventive Science, University of Ferrara, Ferrara, Italy; 4 Centre for Biostatistics, Epidemiology and Public Health, Department of Clinical and Biological Sciences, University of Turin, Turin, Italy

## Abstract

This cross-sectional study assesses the accuracy, sensitivity, and specificity of a large language model used to process unstructured, non-English emergency department (ED) data in medical records.

## Introduction

Timely and accurate identification of injury data from pediatric emergency department (ED) records is critical for injury prevention. However, free text is commonly used in the medical records of EDs in most countries.^1^ In such context, free text is a valuable, and sometimes the only, tool of documentation for epidemiologic surveillance, but its use is challenging.^2^ In this rapidly evolving artificial intelligence era, large language models offer an opportunity to exploit free-text information in medical records. This study evaluated the performance of a large language model in identifying and classifying injury data in Italian from pediatric ED records.

## Methods

The study analyzed 283 468 medical records of the pediatric ED of Padova University Hospital in Padova, Italy, from January 1, 2007, to December 31, 2018.^3^ The Azienda Ospedaliera di Padova Ethics Committee approved this cross-sectional study. Patients signed a written consent form to allow the use of data for scientific purposes. We followed the STROBE reporting guideline.

A subset of the records (n = 40 031) was randomly extracted from the dataset, and the free-text discharge diagnoses in Italian were classified manually by an expert clinician according to the World Health Organization injury classification system.^4^ This manual classification served as the criterion standard for evaluating the Generative Pretrained Transformer 4 (OpenAI) performance of the classification task.

The software manufacturer’s application programming interface end points were used as a basis for the classification task. The large language model was accessed through the openai R package.^5^ The eTable in Supplement 1 presents the prompts used. A description of the classification task methods used is presented in the eMethods in Supplement 1.

The performance of the large language model in the classification task was evaluated by calculating the accuracy, sensitivity, and specificity, which were reported with bootstrap 95% CIs within 1000 iterations. Analyses were conducted using R 4.3.2 (R Project for Statistical Computing).

## Results

The classification task was performed on 8194 records manually classified as unintentional injuries according to the World Health Organization injury classification system. Among the injuries, 520 (6%) were categorized as road traffic, 589 (7%) as falls, 194 (2%) as fires and burns, and 176 (2%) as poisoning. In 12 cases, the injury was drowning; the remaining injuries were categorized under other, which included insect, tick, and animal bites and trauma of undetermined nature (Table 1). Patients with injury included 4325 males (53%) and 3869 females (47%), with a mean (SD) age of 7.3 (4.7) years.

**Table 1.** Sample Characteristics

Characteristic	No. (%) (N = 8194)
Sex	
Female	3869 (47)
Male	4325 (53)
Age	
1-28 d	103 (1)
29 d-1 y	699 (9)
1-3 y	1387 (17)
4-5 y	1390 (17)
6-10 y	1944 (24)
11-15 y	2536 (31)
≥16 y	135 (2)
Nationality	
Italian	4821 (59)
Other^a^	3373 (41)
Injury classification according to the criterion standard	
Drowning	12 (0)
Falls	589 (7)
Fires and burns	194 (2)
Poisoning	176 (2)
Road traffic	520 (6)
Other^b^	6703 (82)

^a^ Other included nationalities of people from all over the world.

^b^ Other injury classification included insect, tick, and animal bites and trauma of undetermined nature.

Performance of the classification task by the large language model was very good (Table 2). The sensitivity was equal to 1.000 points for all categories except for falls (0.997; 95% CI, 0.991-1.000 points). The specificity was at least 0.996. No classification errors were detected for fires and burns and drowning categories.

**Table 2.** Performance Metrics According to Injury Category

Metric	Point estimate (95% CI)				
	Road traffic injuries^a^	Poisoning^a^	Fires and burns^a^	Falls	Drowning^a^
Accuracy	0.996 (0.995-0.997)	0.999 (0.999-1.000)	1.000	0.996 (0.995-0.997)	1.000
Sensitivity	1.000	1.000	1.000	0.997 (0.991-1.000)	1.000
Specificity	0.996 (0.994-0.997)	0.999 (0.999-1.000)	1.000	0.996 (0.995-0.997)	1.000

^a^ No classification errors were observed.

## Discussion

The findings suggest that use of large language models is feasible for processing unstructured free-text information in languages other than English. From a public health perspective, analyzing unstructured information allows for early detection of emerging hazards, helps with identification of injury patterns, and provides data to policymakers for developing preventive measures.

Study limitations include its single-center design and the low prevalence of specific injury mechanisms, requiring assessment of the model’s performance on even larger, preferably multicenter datasets. Despite the potential of large language models in medical research and practice, their use is debated because they pose relevant issues, including ethical concerns, risk of misinformation, and misinformation spread.^6^ However, almost all new technologies come with risks and benefits; what makes the difference is how they are used. Results of this study suggest that large language models are a promising tool for classifying injuries documented in ED records, helping with surveillance.

## Notes

Supplement 1.
eMethods.

eReferences
**eTable.** GPT Prompts for Extraction of Clinical Information

Supplement 2.
Data Sharing Statement
