# Supplementary material for: Use of a Large Language Model to Identify and Classify Injuries With Free-Text Emergency Department Data
Source: JAMA Netw Open. 2024 May 28;7(5):e2413208. doi: 10.1001/jamanetworkopen.2024.13208 (PMC11134210; doi:10.1001/jamanetworkopen.2024.13208)
Supplement: Supplement 1. — eMethods. eReferences eTable. GPT Prompts for Extraction of Clinical Information [file jamanetwopen-e2413208-s001.pdf]

Supplemental Online Content

Lorenzoni G, Gregori D, Bressan S, et al. Use of GPT-4 to identify and classify injuries with free-text emergency department data. *JAMA Netw Open*. 2024;7(5):e2413208. doi:10.1001/jamanetworkopen.2024.13208

eMethods

eReferences

eTable. GPT Prompts for Extraction of Clinical Information

This supplemental material has been provided by the authors to give readers additional information about their work.

© 2024 Lorenzoni G et al. *JAMA Network Open*

eMethods

Reference method: manual classification

Free text discharge diagnoses in the Italian language were classified manually according to the following categories:

1) Injuries Vs. Not injuries
2) Unintentional Vs. Intentional injuries (for injuries only)
3) Type of injury (for unintentional injuries only)

All records were completely anonymous and thoroughly investigated to avoid potential data-disclosure. The records were in the Italian language.

The classification task was performed by an expert clinician following three hierarchical steps. Initially, each diagnosis was classified to determine whether it indicated an injury case. Subsequently, injury cases were classified as either unintentional ("*or so-called "accident" that could have been prevented*" [1]) or intentional. Finally, unintentional injuries were classified according to the World Health Organization classification system [1] of injuries, which identifies five main child injury mechanisms: road traffic injuries, poisoning, falls, fires/burns, and drowning. A sixth category was added and included other types of unintentional injuries.

The manual classification of unintentional injuries served as the gold standard to evaluate the performance of the Generative Pretrained Transformer (GPT)-based classification task.

GPT-based classification

OpenAI application programming interface (API) endpoints were used as a basis for the classification task. The API provides developers with access to advanced artificial intelligence (AI) models, such as GPT, which can be used for a wide range of applications, including Natural Language Processing

© 2024 Lorenzoni G et al. *JAMA Network Open*

(NLP), text generation, translation, summarization, and more. One of the most significant advantages of using the OpenAI API is its ability to perform few-shot learning, where the AI model can quickly adapt to new tasks with minimal training.

GPT models were accessed through the OpenAI API via the openai R package [2], through which users can provide prompts to the trained model, hosted in the cloud, and receive the models' response. In addition to protecting OpenAI's intellectual property, an advantage of this approach is that it completely unburdens users from the prohibitive computational costs of running such a large deep neural network on a local machine. The monetary cost of each request through the API depends on the number of tokens for the prompt and response. A token is the elementary input processed by the model and generally corresponds to a portion of a word (approximately 3/4 characters on average).

When invoking the model, users can specify additional parameters to influence the response provided by GPT. The two most important are 'temperature' and 'presence-penalty', which influence how deterministic the model is when generating its response. In our experiments, we set the penalty at 0.8 and temperature at a value of 0.2 (where lower values correspond to higher determinism in the answer). In fact, our task was to extract specific pieces of information for which there was a single correct answer (high determinism), but we also wanted the model output to be in machine-readable format, which can be directly used for statistical analyses.

We followed the OpenAI's guidelines [3] to generate prompt messages for each of these extractions.

All analyses were performed using R software version 4.3.2 and the openai package [2].

eReferences

1. Branche C, Ozanne-Smith J, Oyebite K, Hyder AA. World report on child injury prevention. Published online 2008.

2. Rudnytskyi I. *Openai: R Wrapper for OpenAI API.*; 2023. https://CRAN.R-project.org/package=openai

3. https://platform.openai.com/docs/guides/prompt-engineering/strategy-split-complex-tasks-into-simpler-subtasks. Last accessed on 25 of January 2024.

© 2024 Lorenzoni G et al. *JAMA Network Open*

eTable. GPT prompts for extraction of clinical information.

Completion sequence	GPT Prompt Text
**#1**	"I will give you the text of a medical case report of a child admitted to an emergency room because of an injury"
**#2**	"The text was derived from a CSV file, with anamnestic and clinical information which are separated by commas"
**#3**	"The text is in Italian"
**#4**	"Answer the following questions by reasoning step by step.<br>First look at the type of the injury and try to classify it."
**#5**	"Clean your answer by using only one of the following categories:<br>- falls<br>- poisoning<br>- road traffic<br>- fires and burns<br>- drowning<br>If you are unable to classify in one of these categories, use the category<br>- other<br>Use only the words corresponding to these categories and use only lowercase. Do not write any other output or comments. What is the type of injury?"

© 2024 Lorenzoni G et al. *JAMA Network Open*
