# Supplementary material for: Use of a Large Language Model to Identify and Classify Injuries With Free-Text Emergency Department Data
Source: JAMA Netw Open. 2024 May 28;7(5):e2413208. doi: 10.1001/jamanetworkopen.2024.13208 (PMC11134210; doi:10.1001/jamanetworkopen.2024.13208)
Supplement: Supplement 2. — Data Sharing Statement [file jamanetwopen-e2413208-s002.pdf]

Data Sharing Statement

Lorenzoni. Use of GPT-4 to Identify and Classify Injuries With Free-Text Emergency Department Data. *JAMA Netw Open*. Published May 28, 2024.
doi:10.1001/jamanetworkopen.2024.13208

Data
Data available: No

Additional Information
Explanation for why data not available: The dataset is available on motivated request to the corresponding author.
